# Dermatofibrosarcoma protuberans of the vulva: margins assessment and reconstructive options – a report of two cases

**DOI:** 10.1186/1477-7819-12-399

**Published:** 2014-12-29

**Authors:** Eduardo Bertolli, Eduard Renè Bretchbuhl, William Ricardo Camarço, Mariane Campagnari, André Sapata Molina, Glauco Baiocchi, Mariana Petaccia de Macedo, Clovis Antonio Lopes Pinto, Isabela Werneck da Cunha, João Pedreira Duprat Neto

**Affiliations:** 1 Skin Cancer Department, AC Camargo Cancer Center, Rua Professor Antonio Prudente 211, 01509-900 São Paulo, Brazil; 2 Gynecologic Oncology Department, AC Camargo Cancer Center, Rua Professor Antonio Prudente 211, 01509-900 São Paulo, Brazil; 3 Pathology Department, AC Camargo Cancer Center, Rua Professor Antonio Prudente 211, 01509-900 São Paulo, Brazil

**Keywords:** DFSP, Vulva, CCPDMA, Reconstruction, Surgical oncology

## Abstract

**Background:**

Dermatofibrosarcoma Protuberans (DFSP) of the vulva is rare and oncologic surgery with free margins may lead to severe functional damage, requiring multidisciplinary approach regarding resection, margin assessment and reconstruction.

**Case Report:**

Two cases of DFSP in vulva were treated in a single institution. A 28-year-old patient with an incisional biopsy in the vulvar region revealing DFSP underwent a partial vulvectomy with clitoris preservation. Pathological studies revealed free margins and reconstructive surgery was performed. This patient is disease free in a 40 months follow up. The other, a 57-year-old patient was also referred after an incomplete resection of a DFSP in the vulvar region. A 1-cm margim resection followed by Complete Circumferential and Peripheral Deep Margin Assessment (CCPDMA) was performed. Although the upper lateral margin was positive, it was possible to perform another wide local excision with preservation of the clitoris and primary closure.

**Conclusion:**

DFSP of the vulva requires an accurate evaluation of margins, resections following oncological principles and reconstruction. Although being a very challenging lesion that usually implies difficult surgical management, if treated in a multidisciplinary environment, with surgical oncologists, experienced dermatopathologists and reconstructive surgeons can achieve good results. Even in difficult cases that presents with large lesions and compromising challenging areas, a complete oncologic resection can be performed minimizing functional damage for the patient.

**Electronic supplementary material:**

The online version of this article (doi:10.1186/1477-7819-12-399) contains supplementary material, which is available to authorized users.

## Background

Dermatofibrosarcoma protuberans (DFSP) is a rare skin mesenchymal tumor that usually affects the trunk and extremities. Vulvar DFSP is rare, with fewer than 40 cases previously reported in the literature [1, 2]. Surgery has been reported as the main therapeutic option for DFSP treatment. However, there is no consensus on margin assessment protocols. The most frequently used techniques are Mohs surgery, wide local excision (WLE) with 2.0 or 3.0 cm margins and surgery followed by three-dimensional complete circumferential and peripheral deep margin assessment (CCPDMA) [3–6].

The CCPDMA technique consists of excising the tumor with a margin smaller than the traditional 3.0-cm one. The specimen is submitted to conventional histologic processing and margins are completely evaluated by the pathologist. Additional study with immunohistochemistry (IHC) may be useful for margin assessment in some doubtful cases. This technique is associated with a more accurate evaluation of the margins and literature reports rates of recurrence lower than 10% [5, 7].

Another very important aspect of surgery in vulvar DFSP is the clitoris preservation and reconstruction after the lesion is removed, because this directly affects sexuality and quality of life of these patients [8, 9]. Better results are achieved with a multidisciplinary approach. The objective of this study was to present two cases of vulvar DFSP managed by the Skin Cancer and Gynecology Oncology Departments of AC Camargo Cancer Center with minimal functional damage for the patient. This case report was approved for publication by Fundação Antonio Prudente ethics committee.

## Case presentation

**Figure 1:**
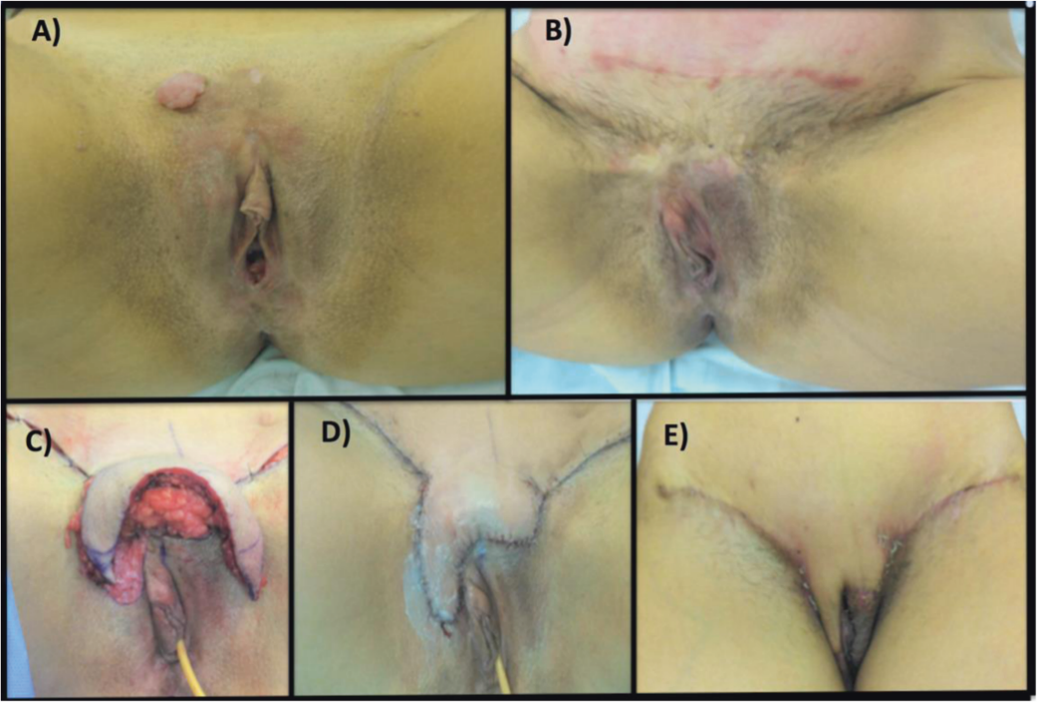
**Patient 1. (A)** Clinical aspect of the residual lesion of dermatofibrosarcoma protuberans of the vulva before the surgery. **(B)** One year after the first surgery, a skin expander was placed. **(C)** The expander was taken out and the inferior 2/3 of the flap was split. **(D)** the distal portion was incised horizontally on both sides and folded after, in order to reconstruct the labius majoras. **(E)** Follow up after 8 weeks.

**Figure 2:**
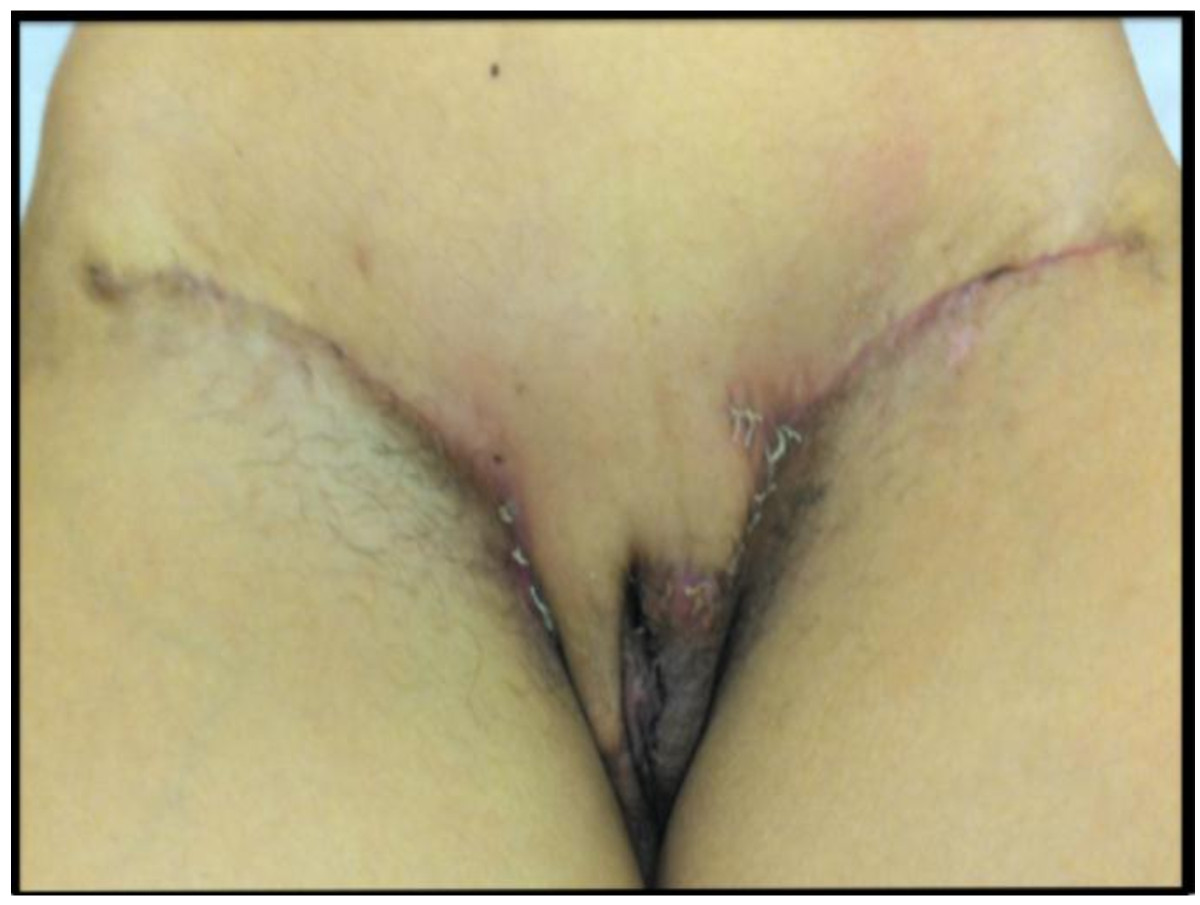
**Patient 2: final clinical aspect of the second surgery when primary closure was performed twice.**

The first case is a 28-year-old female patient referred to us after an incisional biopsy in the vulvar region revealing DFSP. She presented to us with a 5.0-cm lesion in the mons pubis (Figure 1A). Surgery was indicated, and consisted of a vulvectomy with a local advancement flap as the first approach in order to obtain a 2.0 cm margin from the tumor. Margins were assessed by CCPDMA protocols and they were all free of disease. Therefore, it was possible to avoid clitoris resection.To perform the vulvar reconstruction, a skin expander was placed in the inferior part of the abdominal wall one year after vulvectomy (Figure 1B). Weekly expansion was performed 3 weeks after the procedure and, in the third month, sufficient skin was attained in this area. The expander was taken out and the inferior 2/3 of the flap was split. The skin of the mons pubis was decorticated and the medium and proximal portions of the flap were advanced to protect the pubis bone. The distal portion was incised horizontally on both sides and folded after, in order to reconstruct the labius majoras (Figure 1C and D).This patient has been followed for almost 40 months (Figure 1E). There is no clinical or radiological evidence of recurrence. Regarding the surgery, we considered that a very adequate aesthetic effect was achieved. The patient verbally reported good quality of life, although no quality of life (QOL) questionnaires were applied.The second case is a 57-year-old female patient, also referred to our hospital after an incomplete resection of a DFSP in the vulvar region. She presented to us with a 2.0 cm residual lesion in the right labium major. As the lesion was too close to the clitoris, we performed a 1.0-cm margin instead of the traditional 2.0-cm one, followed by primary closure. The CCPDMA revealed all positive margins except for the upper lateral region of the specimen. The patient was submitted to another WLE of 2.0 cm and subsequent primary closure could be performed once more (Figure 2A). This patient has been followed for 10 months without any aesthetic or functional issue related to the surgery.

## Conclusion

Regardless being a very rare disease, DFSP in the vulva represents a very challenging situation for the oncologic and plastic surgeons. The resection must follow oncological principles, which sometimes may lead to several aesthetic and functional impacts for the patient due to the difficulty in planning how wide the resection should be [4]. In this context, reconstructive surgery plays a fundamental role in the management of DFSP patients.

There are just few cases of vulvar DFSP reported in the literature. See Table 1 for a summary of previously reported cases of DFSP in the vulva with clinical, treatment and follow-up information. The largest series, from the MD Anderson Cancer Center, reported 13 cases. All of the six cases with positive margins after resection developed tumor recurrence. There was only one relapse between the cases with negative margins after WLE that occurred in a patient showing fibrosarcomatous transformation in the recurrent tumor [1].

**Table 1:** **Summary of the reported cases of Dermatofibrosarcoma Protuberans of the vulva with clinical, treatment and follow up information**

Author	Cases	Age, years	Clinical presentation	Size, cm	Initial treatment	Outcome/follow up
Edelweiss and Malpica [1]	1	36	Right labium major mass	5	Excisional biopsy	LR at 12 months. AWD at 16 months
Edelweiss and Malpica [1]	2	57	Right labium major mass	3	Excisional biopsy	LR at 60 months, then WLE (2x) until negative margin. NED at 216 months.
Edelweiss and Malpica [1]	3	69	Mons pubis mass	3	Excisional biopsy + WLE	Dead of other causes at 144 months
Edelweiss and Malpica [1]	4	48	Left paraclitoral mass	1.2	Excisional biopsy + WLE	LR, then WLE. NED at 144 months.
Edelweiss and Malpica [1]	5	46	Right labium major pigmented lesion	4	Excisional biopsy + WLE (x2)	NED at 84 months.
Edelweiss and Malpica [1]	6	76	Right labium major and crural fold mass	15	Excisional biopsy + WLE	LR at 7 months, then WLE with negative margin. At 14 months, metastasis to chest wall. At 18 months, metastasis to hip and thigh. Death of the disease at 34 months.
Edelweiss and Malpica [1]	7	44	Left labium major mass	4	Excisional biopsy + WLE	NED at 53 months
Edelweiss and Malpica [1]	8	39	Right labium major mass	N/A	WLE	LR at 48 months. Then, WLE with positive margin. At 60 months, local recurrence. Then WLE with positive margin. Used Gleevec. NED at 72 months
Edelweiss and Malpica [1]	9	30	Right labium major and mons pubis mass	N/A	Excisional biopsy + WLE	LR at 11 months. Then, WLE. AWD at 24 months
Edelweiss and Malpica [1]	10	23	Right labium major mass	4	Excisional biopsy + WLE	NED at 2 months
Edelweiss and Malpica [1]	11	30	Left labium major mass	N/A	Excisional biopsy	LR at 60 months followed by WLE with positive margin. Then, partial vulvectomy with negative margin. NED at 96 months
Edelweiss and Malpica [1]	12	44	Right labium major mass	2.5	Excisional biopsy + radical vulvectomy	NED at 36 months
Edelweiss and Malpica [1]	13	58	Paraclitoral mass extending into vagina	4.8	WLE	NED at 3 months
Soltan [10]	14	83	Left labium major/minor nodule	5	Excisional biopsy	N/A
Agress *et al*. [11]	15	56	Left labium major	N/A	Excisional biopsy	LR, then WLE
Bock *et al*. [12]	16	52	Right mons pruritus	8	Excisional biopsy	LR, then WLE
Barnhill *et al*. [13]	17	42	Right vulva lateral to clitoris nodule	1	Excisional biopsy	LR, then WLE and hemivulvectomy
Leake *et al*. [14]	18	37	Left mons and labium major nodule	6.2	Excisional biopsy	LR, then wide radical excision
Leake *et al*. [14]	19	59	Right labium major nodule	5	Excisional biopsy	LR, then partial radical vulvectomy
Panidis *et al*. [15]	20	30	Right labium major nodule	2	WLE	LR, then radical vulvectomy
Aartsen and Albus-Lutter [16]	21	50	N/A	1.2	Radical vulvectomy	N/A
Karlen *et al*. [17]	22	36	Left labium major irritated lump	5	WLE	N/A
Nirenberg *et al*. [18]	23	41	Left labium major lump	8	WLE	N/A
Alverez-Canas [19]	24	58	Left labium major mass	3.2	WLE	N/A
Soergel *et al*. [20]	25	47	Left vulva mass + lung metastasis	3	Partial radical vulvectomy + chemotherapy	LR, then WLE + radiotherapy
Ghorbani *et al*. [21]	26	47	Left paraclitoral area	N/A	Hemivulvectomy	LR, then wide radical excision
Ghorbani *et al*. [21]	27	44	Left labium major mass	4	Wide radical excision	N/A
Ghorbani *et al*. [21]	28	66	Mons mass	1.5	WLE	N/A
Ghorbani *et al*. [21]	29	36	Right labium mass	5	Excsional biopsy	N/A
Moodley and Moodley [22]	30	39	Left labium major mass	12	WLE	N/A
Vanni *et al*. [23]	31	39	Inferior vulva/perineum mass	6	WLE	N/A
Kholova *et al*. [24]	32	31	Left labium major recurrent nodule	1.5	Excisional biopsy	LR, then WLE + radiotherapy
Ohlinger *et al*. [25]	33	36	Left vulva nodule	2.8	Excisional biopsy	LR, then WLE
Hancox *et al*. [26]	34	55	Right labium major mass	8	MMS	NED
Hammonds and Hendi [6]	35	59	Right labium major mass	4	MMS	NED
Doufekas *et al*. [3]	36	39	Left labium major nodule	N/A	Incisional biopsy	MMS with primary closure. NED at 3 years
Zizi-Sermpetzoglou *et al*. [27]	37	66	Mons pubis	N/A	WLE	NED

N/A, not available; WLE, wide local excision; MMS, Mohs micrographic surgery; LR, local recurrence; AWD, alive with disease; AWOD, alive without disease; NED, no evidence of disease).

Based on this paper, the role of an adequate assessment of margins in these cases can be assumed. In our service, the protocol is to submit the specimen to conventional histologic processing with complete evaluation of the margins by CCPDMA by the pathologist, and IHC study whenever necessary [2, 5]. The morphologic and molecular pathologic findings of vulvar DFSP are quite similar to DFSP in other sites, including the frequent finding of the fusion between collagen type I alpha 1 gene (COL1A1) on chromosome 17 and the platelet-derived growth factor B-chain (PDGFB) gene on chromosome 22 [23, 28].

There is also scarce literature on reconstructive options in vulvar DFSP. There are two recent reports on Mohs surgery for vulvar DFSP, and both were submitted to primary closure [3, 6]. No data related to clitoris preservation or major vulvar reconstructions have been found.

Skin expansions are often used to reconstruct burned areas or breasts after a mastectomy or to hide scars [29]. Although there is no strong evidence in the literature to supports its use in skin tumors, we believe that it can be safely used since the tumor resection is not delayed because of the use of the expansion. Also, in our case we have had a very rigorous assessment of the pathological margins to ensure that the resection was complete. Moreover, the patient was kept for over a year in clinical follow up before starting the expansion.

Our first case illustrates an example of a major vulvar reconstruction using skin expansion followed by a skin and fat tissue flap. Although it has been done in only one patient, the clinical outcome suggests this can be a feasible option in such cases. Both our patients were submitted to clitoris-sparing surgery, which was only possible due to the utilization of the CCPDMA protocol by our Pathology Department.

We conclude that vulvar DFSP, although a very challenging lesion that usually implies difficult surgical management, if treated in a multidisciplinary environment with surgical oncologists, experienced pathologists and reconstructive surgeons, can achieve good results. And even in difficult cases that present with large lesions and compromising challenging areas, a complete oncologicresection can be performed, minimizing functional damage for the patient.

## Authors’ original submitted files for images

Below are the links to the authors’ original submitted files for images. Authors’ original file for figure 1 Authors’ original file for figure 2

## Abbreviations

DFSP: dermatofibrosarcoma protuberans
WLE: wide local excision
CCPDMA: 3-dimensional complete circumferential and peripheral deep margin assessment
IHC: immunohistochemistry
COL1A1: collagen type I alpha 1
PGDF: Platelet-derived growth factor B-chain.
