# Supplementary figures and images for: Dermatofibrosarcoma protuberans of the vulva: margins assessment and reconstructive options – a report of two cases
Source: World J Surg Oncol. 2014 Dec 29;12:399. doi: 10.1186/1477-7819-12-399 (PMC6389240; doi:10.1186/1477-7819-12-399)

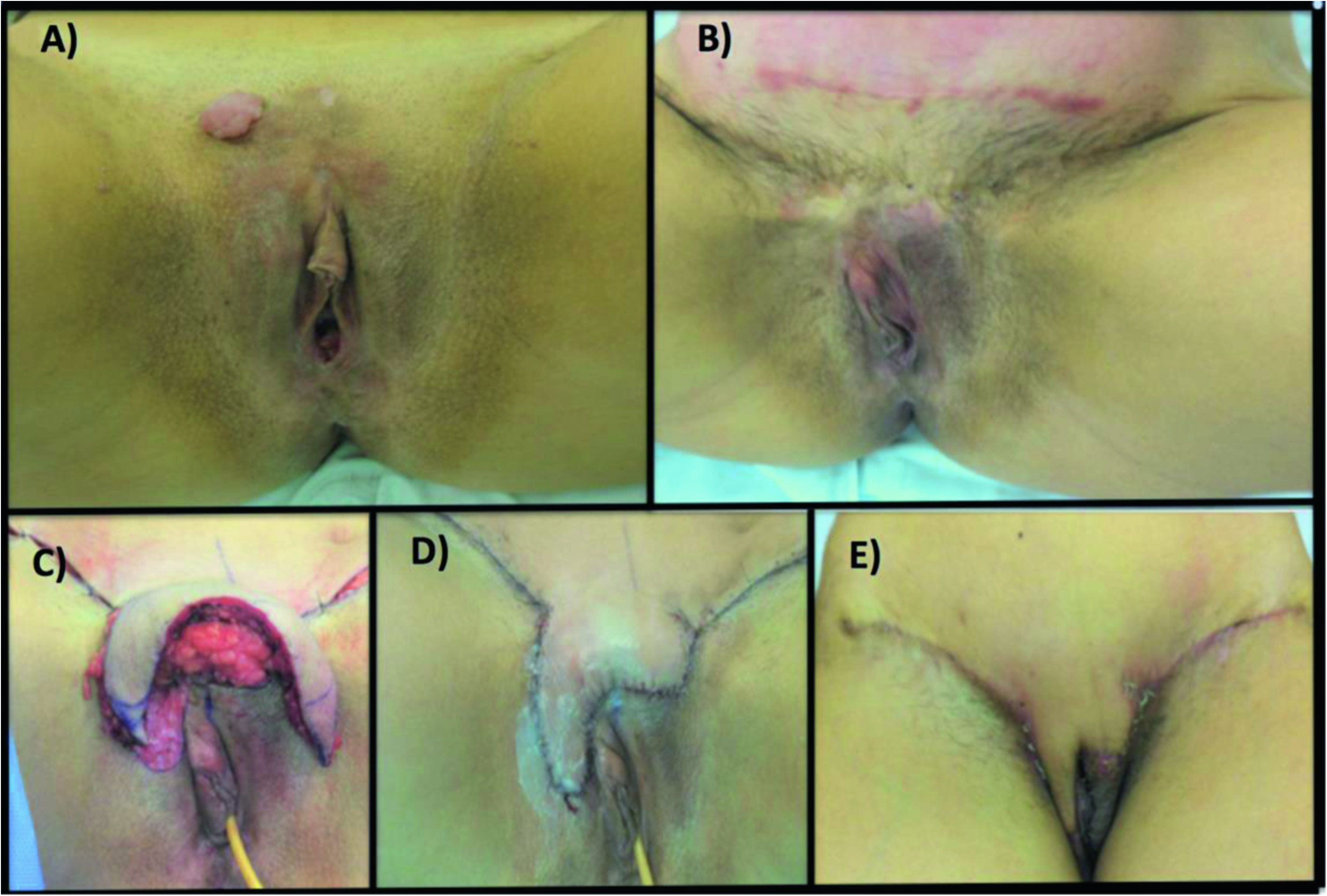

Supplement: Supplementary file 1 — Authors’ original file for figure 1 [file 12957_2014_1919_MOESM1_ESM.tif]

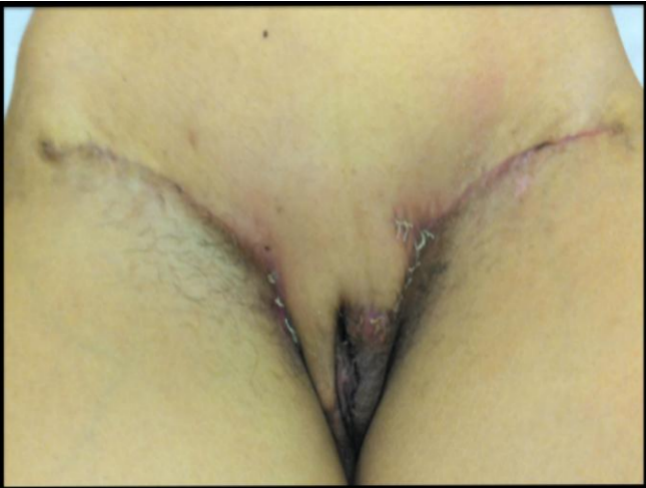

Supplement: Supplementary file 2 — Authors’ original file for figure 2 [file 12957_2014_1919_MOESM2_ESM.pdf]
